# The role of the LRRK2 gene in Parkinsonism

**DOI:** 10.1186/1750-1326-9-47

**Published:** 2014-11-12

**Authors:** Jie-Qiong Li, Lan Tan, Jin-Tai Yu

**Affiliations:** Department of Neurology, Qingdao Municipal Hospital, School of Medicine, Qingdao University, No. 5 Donghai Middle Road, Qingdao, 266071 PR China

**Keywords:** LRRK2, Parkinsonism, Pathology, Clinical features, Pathogenic mechanism

## Abstract

Parkinson’s disease (PD), like many common age-related conditions, has been recognized to have a substantial genetic component. Multiple lines of evidence suggest that Leucine-rich repeat kinase 2 (LRRK2) is a crucial factor to understanding the etiology of PD. LRRK2 is a large, widely expressed, multi-domain and multifunctional protein. *LRRK2* mutations are the major cause to inherited and sporadic PD. In this review, we discuss the pathology and clinical features which show diversity and variability of LRRK2-associated PD. In addition, we do a thorough literature review and provide theoretical data for gene counseling. Further, we present the evidence linking *LRRK2* to various possible pathogenic mechanism of PD such as α-synuclein, tau, inflammatory response, oxidative stress, mitochondrial dysfunction, synaptic dysfunction as well as autophagy-lysosomal system. Based on the above work, we investigate activities both within GTPase and outside enzymatic regions in order to obtain a potential therapeutic approach to solve the *LRRK2* problem.

## Introduction

Parkinson disease (PD), also known as shaking palsy, is a common neurodegenerative disorder clinically characterized by stilly shacking, bradykinesia, rigidity muscles and abnormal posture and pace which were first systematically described by an English doctor named James Parkinson. The neuropathological hallmarks are characterized by a progressive loss of dopaminergic neurons in the substantia nigra pars compacta (SNpc) and the presence of proteinaceous inclusions immunoreactive for α-synuclein termed as Lewy bodies and dystrophic Lewy neurites in surviving neurons. Traditionally, PD has been considered a sporadic neurodegenerative disorder. However increasing evidences of family aggregation indicate that there may be a link between PD and gene.

**Figure 1:**
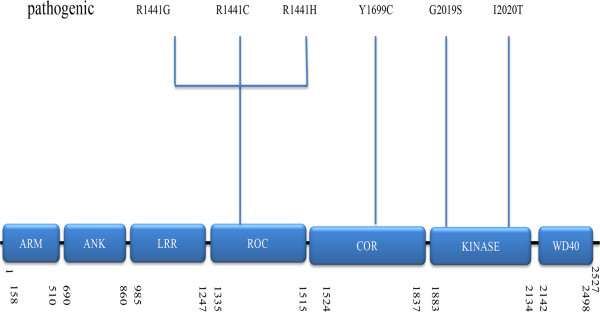
**Schematic representation of LRRK2 and the pathogenic mutations associated with PD ARM, Armadillo; ANK, Ankyrin repeat; LRR, leucine-rich repeat; ROC, Ras of complex proteins: GTPase; COR, C-terminal of ROC; WD40, WD-40 domain.** Potential pathogenic mutations are shown together.

To date, in addition to *α-synuclein* (*SNCA*), *parkin* (*PARK2*), *UCH-L1* (*PARK5*), *PINK1* (*PARK6*), *DJ-1* (*PARK7*), *LRRK2* (*PARK8*), and *ATP13A2* (*PARK9*), there are also GBA, VPS35, EIF4G1, PARK16 which have been isolated to be responsible for PD based on family based linkage analysis. Among all the causative genes, mutations in *α-synuclein* and *LRRK2* have been proved to associate with autosomal dominant (AD) PD, while mutations in *parkin*, *PINK1*, *DJ-1*, and *ATP13A2* are linked to autosomal recessive PD. Mutations in LRRK2 are the most common genetic cause of both familial and sporadic PD [1]. The product of this gene, also known as dardarin, is a highly conserved large 286-kDa protein that contains multiple, independent domains belonging to the ROCO protein family. To date, only six of 20 mutations of this gene have been demonstrated to be pathogenic (Figure 1), and the most common mutation of LRRK2 gene is G2019S (accounting for 5–6% of familial PD, and in 1–2% of sporadic cases), whose importance lies not only in its relatively high frequency in specific populations but also its association with late-onset sporadic ‘classical’ PD [2].

Based on the previous fundamental researches on *LRRK2*, the possible mechanism of LRRK2 participating in the pathogenic of parkinsonim will be introduced in this review.

## The structure and physiological functions of LRRK2

LRRK2 is an unusually large protein (2527 amino acids) classified as a member of the ROCO superfamily which is characterized by the presence of tandem Ras of complex (Roc) G-domain, kinase domains and carboxy- terminal of Roc (COR) sequence which links them. The high conservation of the ROC–COR (ROCO) module throughout evolution suggests the close functional interdependence of the two domains [3]. In Roco family members, the COR domain always occurs in tandem with the G-domain. The COR domain consists of two parts: the highly conserved N-terminal part that interacts with the Roc domain and the less conserved C-terminal part functions as dimerization device [4]. In addition, domains related to protein–protein interaction include armadillo (ARM), ankyrin (ANK), leucine-rich repeats (LRR) and WD40 domains. ANK domain compromising 7 ankyrin-type repeats and 13 leucine-rich repeats (LRR) have been identified in the N-terminal region of LRRK2 [5] while WD40 domain is consisted by C-terminal repeats [6]. (Figure 1) In addition, whether the armadillo domain is a part of the N-terminal has been controversial, but recent studies conclude that the answer is affirmative [3].

The physiological functions of LRRK2 can be demonstrated from its two distinct enzymatic domains: the kinase domain could catalyze phosphorylation and the ROC-GTPase domain is involved in GTP-GDP hydrolysis. A recent study showed that disrupting kinase activity reduced mutant LRRK2 toxicity by affecting LRRK2 levels rather than kinase activity per se [7]. In addition to those enzymatic functions, multiple protein–protein interaction regions indicate that LRRK2 may function as a scaffolding protein contributing to the formation of a multiprotein signaling complex [8]. Although the precise physiological function of LRRK2 remains largely unknown, recent studies have indicated that LRRK2 at least is involved in cellular functions such as neurite outgrowth, cytoskeletal maintenance, vesicle trafficking, autophagic protein degradation, and the immune system [9]. Moreover, the experience in rat neurons suggested a role of LRRK2 in the regulation of neurite process morphology.

## Mutations associated with PD in LRRK2

In order to study the actual mechanism of PD caused by *LRRK2* mutations, extensive effort has been devoted to the investigation of the mutation of this gene. Up to now, established dominant inherited PD­associated mutations in *LRRK2* include p.G2019S, p.R1441C/G/H, p.Y1699C, p.I2020T and p.N1437H.

Among them, G2019S is especially frequent in PD patients, accounting for 13% of Ashkenazi Jewish and 30% of Arab­Berber populations respectively. It also contributes to 1–7% of familial cases of parkinsonism of European and US origin and 1–3% of sporadic PD [1]. More specifically, G2019S was found in 5.6% of North American families with familial PD, 6.6% of families with AD PD from Italy, Brazil and Portugal and 1.7% of British patients with sporadic PD. Moreover, this mutation accounts for 0.7% of the sporadic cases and 7.7% of the AD cases of PD in Russian population [10]. There have been reports that p.G2019S, which is found in KIN domain, and has been demonstrated to give rise to a hyperactive kinase, increases autophosphorylation and phosphorylation of generic substrates [11]. There are two plausible explanations for this; introducing a phosphorylation site (most of the mutations introduce an S or a T) or stabilizing active or inactive forms by hydrogen bonds [5].

Nevertheless, other group could not detect any significant changes. This discrepancy might be related to specific effects given the substrate used by Liu et al. [12]. In addition to p.G2019S mutation, there are two missense variants (R1628P and G2385R) which are associated with susceptibility to PD in Han Chinese and East-Asians. The LRRK2 variant p.R1628P and p.G2385R are unlikely to cause PD, but increase risk for disease by about 1.84 fold and 2 fold respectively [13].

## Animal models of LRRK2 Parkinsonism

Animal models have been widely used in the research of *LRRK2*. Overexpression of *LRRK2* in Drosophila leads to age-dependent DA-responsive reductions in locomotor activity and loss of DA neurons [14–16]. In a similar manner, overexpression of *LRRK2* results in degeneration of DA neurons in C. elegans [17]. Compared with p.G2019S mutants or nontransgenic controls, the expression of human WT LRRK2 in the nematode worm C. elegans results in a longer lifespan [18]. Since Drosophila and C. elegans do not express a-synuclein and a prominent feature of PD caused by mutations in LRRK2 is a-synuclein pathology, the utility of studying mechanisms of LRRK2 neurodegeneration in flies and worms is potentially problematic.

In contrast to invertebrate models, current transgenic mouse models are not very robust PD models. Transgenic mice, using bacterial artificial chromosome (BAC) expressing WT LRRK2, or LRRK2 with p.R1441G or p.G2019S mutations, have minimal evidence of neurodegeneration [19]. Conditional expression of WT or p.G2019S LRRK2 by incorporating the calcium/calmodulin-dependent protein kinase IIa (CamKII) promoter also failed to induce degeneration of dopaminergic neurons [20]. Additionally, when the p.R1441C mutation is expressed under the control of the endogenous regulatory elements, by knockin of the p.R1441C mutation, there is no degeneration of DA neurons [21]. Although gross neuronal death was not found, most of current LRRK2 transgenic mice had abnormalities in the nigrostriatal system, including decreased dopamine release or behavioral deficits, which are DA responsive. In contrast to the other LRRK2 transgenic models, the LRRK2 p.R1441G BAC transgenic mice that have progressive age-dependent locomotion deficits are levodopa and apomorphine responsive, although there is still no substantial neurodegeneration identified [19].

## Neuropathology of *LRRK2*-associated PD

There is a wide pathological spectrum associated with *LRRK2* mutations, and the same mutation can cause quite diverse neuropathology (Table 1) [22–25]. Some are characterized by hyperphosphorylated tau or ubiquitin-immunoreactive inclusions. However, more cases show pathological characteristics of the presence of Lewy bodies, Lewy neuritis and substantia nigra neuronal loss [26]. Those pathological characteristics among different mutations could manifest either as intercombination or just as separation. Postmortem analyses of PD patients with neurodegeneration related to *LRRK2* mutations have demonstrated both with and without Lewy pathology. Therefore, further studies comparing *LRRK2* mutation cases with these two adverse pathological types are expected to help elucidate the contribution of insoluble α-synuclein aggregates to neurodegeneration in these genetic forms of PD. As such, obtaining a enough large number of well-described neuropathological cases of *LRRK2* and other genetic forms of PD may be required to explain why certain gene mutations are associated with Lewy pathology, whereas others without it, and still others with heterogeneous pathologic features [27].

**Table 1:** **Characteristics of LRRK2 substitutions associated with parkinsonism**

Amino-acid variation	Position	Protein domain	Main phenotype	Risk ethnicty	Neuropathology	Ref
**R1441C**	Exon31	ROC^c^	PD	Middle European	Lewy bodies	[22]
					SN neuronal loss	
					Neurofibrillary tangles	
					Ubiquitin staining	
**R1441G**	Exon31	ROC^c^	PD	Caucasian (Basque Country) Italy	SN neuronal loss	[28]
					Ubiquitin staining	
**R1441H**	Exon31	ROC^c^	PD	NA^b^	Unknown	[23]
**R1628P**	Exon 34	COR^c^	PD	Chinese	Unknown	[24]
**Y1699C**	Exon 35	COR^c^	PD	NA^b^	Lewy bodies	[25]
					SN neuronal loss	
					Neurofibrillary tangles(Stage II^a^)	
					Ubiquitin staining	
**G2019S**	Exon 41	Kinase	PD	In virtually all the populations especially North African	Lewy bodies	[30, 32, 43]
					SN neuronal loss	
					Neurofibrillary tangles (Stage V^a^)	
					Ubiquitin staining	
**I2020T**	Exon 41	Kinase	PD	NA^b^	Lewy bodies	[29]
					SN neuronal loss	
**R2385R**	Exon 48	WD40	PD	East Asian	Unknown	[13]

^a^Stage refers to the highest reported Braak stage.

^b^ *NA*, Not applicable.

^c^ *Roc*: Ras of complex (GTPase). *COR*: C-terminal of Ras.

Lewy bodies (LB) is the most typical and widely spread pathology in *LRRK2* parkinsonism which restricted to the brainstem, the cortex and limbic system, or in the case of pure nigral degeneration may be absent altogether [2]. The wide expression of LRRK2 in brain tissue and the location to LBs in the brainstem, suggest that they are in the same pathway. Besides, along with the tauopathy detected in many cases, there is further evidence that LRKK2 mutations may up-regulate a number of critical pathways, ultimately resulting in cell death [2]. Autopsies from four p.R1441C carriers have been reported that cell loss and gliosis in the substantia nigra were found in all the cases. Among them, two cases had Lewy body disease (LBD) or diffuse LBD with the rest had no distinctive pathology and the last case with tangle pathology in the absence of LBD. As to p.R1441G substitution, there is one autopsy report that showed isolated neuronal loss in the substantia nigra without LBD pathology [28]. However, no pathology reports are available for p.R1441H substitution carriers. Two families with p.Y1699C substitution carriers showed neuronal loss with depigmentation and gliosis in the substantia nigra in one case and ubiquitin-immunoreactive intranuclear and cytoplasmatic inclusion in the other. The neuropathology of p.I2020T mutation was showed to be pure nigral degeneration at the beginning. However, recent report has identified one patient with LBD and one patient with glial cytoplasmic inclusions (GCIs) whcich highlights the pleomorphic pathology in *LRRK2* parkinsonism [29].

It is perplexing that how one identical mutation in the same kindred can generate different pathologies. For the reason of this occurrence, there are several hypotheses, which are not mutually exclusive. For example, the genetic variation at other loci such as MAPT and SNCA which encode the protein tau and α-synuclein respectively could be the obvious candidates to influence pathologies. In addition, different environmental factors such as pesticides and head trauma might steer LRRK2 either towards tau or α-synuclein pathology. However, the certain link still needs large numbers of cases of LRRK2 PD with neuropathological correlation. Moreover, the relative balance between an LRRK2 gain of function (via kinase dysregulation and aggregation of proteins) and loss of function (via ERK pathway inactivation or downregulation of protein transcription) determines different pathologies.

## Clinical features of LRRK2-associated parkinsonism

Clinical features resemble those of late-onset sporadic PD, bradykinesia, rigidity, tremor, and good L-dopa response which have been confirmed by clinical and positron emission tomography (PET) studies. However, female patients carrying the p.G2019S mutation had an age of onset 10 years earlier than the male carriers. Although earlier studies considered tremor as the predominant feature of *LRRK2* carriers, in a recent study, tremor was observed less in patients with LRRK2 p.G2019S than in idiopathic PD [30]. Analyzing the results of the LRRK2 p.G2019S mutation in a large cohort of Russian patients with sporadic and familial PD, we found that all patients carrying the LRRK2 p.G2019S exhibited typical levodopa-responsive parkinsonism, with asymmetric onset of symptoms and variable combination of bradykinesia, rigidity and rest tremor [31]. Moreover, postural tremor was a prominent and early feature. Postural instability and dystonia (including unilateral foot dystonia as a presenting symptom) were rare, and cognitive abnormalities and autonomic dysfunction were absent. There was also Levodopa-induced dyskinesia in one patient who carries both the LRRK2 p.G2019S and parkin exon 5 duplication: this was marked peak-dose dyskinesia of choreic type involving extremities (with gait being almost impossible) and accompanied by dystonic anterocollis [10]. Notably, there was variability in ages of the disease onset in this cohort, ranging from 39 to 71 years. Intending to explain this variability, studies have been conducted in the patients with APOE gene showing that all the patients were carrying two copies of the most common allele, APOE-ϵ3. Consequently, this variability of onset ages could not be explained by genotypes of the APOE gene [10]. In addition, the phenotype of LRRK2 p.G2019S mutation in the Argentinean cohort was indistinguishable from patients with idiopathic PD [32]. Moreover, multiple system atrophy (MSA) is characterized clinically by parkinsonism, however, p.G2019S mutation in the *LRRK2* gene is unlikely to be associated with MSA [33].

In addition to the common p.G2019S carriers, the present study highlights that the distribution in age at onset and clinical features in LRRK2 p.R1441C patients are similar to idiopathic and LRRK2 p.G2019S parkinsonism, indicating that the effect of mutations in different domains of the LRRK2 protein lead to similar phenotypes [34]. LRRK2 p.R1441G transgenic mice show decreased locomotors activity which is considered the hallmark of motor symptoms of PD in human patients. They also display gastrointestinal dysfunction at an early stage but do not have abnormalities in fine behaviors, olfaction, pain sensitivity, mood disorders and learning and memory compared to the controls [35]. As to patients carrying the p.Y1699C mutation, mainly from a British kindred, showed initially symptoms of asymmetrical rest tremor, and then bradykinesia, rigidity, and postural instability with good reactivity to levodopa treatment [36]. Unilateral leg tremors at onset and foot dystonia are prominent features in p.Y1699C patients. However, PET scan analysis indicated that in vivo neurochemical phenotype associated with these two mutations is indistinguishable from that of sporadic PD.

However, to date, most genetic screens have been limited to PD clinic populations, with only a few studies referring to other neurodegenerative diseases. *LRRK2* mutation has been assumed to play an upstream influence on the etiology of not just PD but also several α-synuclein and tau pathologies, including Alzheimer disease (AD) [37]. A research reported two *LRRK2* mutation carriers who showed cognitive impairment, leading to clinical diagnoses of corticobasal syndrome (CBS) and primary progressive aphasia (PPA), a subtype of frontotemporal dementia (FTD). The diagnoses here are clinical, with as yet no pathologic confirmation. The radiographic pattern, however, is consistent with patterns of atrophy in CBS reported, and the Cerebrospinal Fluid (CSF) findings of normal to low tau and normal amyloid-beta 42 (Aβ42) corroborate the diagnosis of a non-AD dementia [38]. Another research conducted among symptomatic *LRRK2* patients, sporadic Parkinson’s disease patients as well as asymptomatic *LRRK2* mutation carriers and matched healthy controls aim to evaluate a possible endophenotype in LRRK2-associated PD. Compared to sporadic Parkinson’s disease patients, *LRRK2* patients had an earlier onset of motor symptoms and a more benign phenotype of motor and non-motor characteristics. Moreover, depression scores were higher in LRRK2 patients. The younger age at onset and the awareness of the mutational status and of the risk to pass the mutation to offspring may be taken into consideration to be in charge of these mood disturbances. In contrast, mood disorder susceptibility genes may modify *LRRK2* mutation penetrance. Either relationship would have profound implications for PD screening and treatment [39]. In addition, no clinical differences were found regarding motor and non-motor symptoms in asymptomatic *LRRK2* mutation carriers in comparison to controls. Transcranial sonography showed hyperechogenicity of the substantia nigra in both patients’ cohorts and asymptomatic *LRRK2* mutations carriers [40].

Other than typical symptoms of PD, there are also symptoms belonging to parkinsonism yet to be examined to have connection to *LRRK2*. Analysis of 14 *LRRK2* mutations in a cohort constituted by patients who were diagnosed with late-onset (age of onset ≥ 56 years old) sporadic PD and other parkinsonian disorders such as progressive supranuclear palsy (PSP), multiple system atrophy (MSA), corticobasal ganglionic degeneration (CBGD), and other atypical parkinsonism (AP) syndromes demonstrated that variants are rare among these Parkinson’s Plus disorders, suggesting that, despite the pleomorphic pathologies in LRRK2-positive patients, routine LRRK2 screening in non-PD parkinsonian disorders may not be cost-effective. More data on the prevalence of *LRRK2* mutations in Parkinson’s Plus syndromes, and exact age-dependent penetrance for each mutation in sporadic and familial PD patients in different ethnic populations will be required [41].

## Genetic counseling

Genetic counseling has been defined as a communication process which deals with the human problems associated with the occurrence, or risk of occurrence, of a genetic disorder in the family. Much attention has been paid on familial aggregation of PD and there have been descriptions of kindreds in which parkinsonism appears to follow a mendelian pattern of inheritance [42]. Mutation in *LRRK2* has a close relationship with PD to which clinical assessments and detailed histories in patients with hereditary PD should be applied with caution in the diagnosis and counseling of patients.

Researchers from 21 centers across the world collaborated to study the risk of PD for individuals who inherit or are at risk of inheriting a deleterious mutation in LRRK2, showing that the frequency of the LRRK2 p.G2019S mutation was 1% for patients with sporadic PD and 4% of patients with hereditary PD. Notably, the frequency was highest in the Middle East and higher in southern Europe than in northern Europe. Therefore, careful clinical assessments and detailed histories should be taken for patients within this population. This prevalence is similar to those for other neurological disorders, such as multiple system atrophy (4 per 100 000), progressive supranuclear palsy (6 per 100 000), motor neuron disease (6 per 100 000), and common single-gene disorders, such as Huntington’s disease (2 per 100 000) and hemophilia A (5 per 100 000). Moreover, 3 cases per 100 000 is an underestimation of the overall prevalence of mutations in LRRK2. Moreover, comparing with patients with idiopathic PD patients, patients with *LRRK2* G2019S had a lower risk of cognitive impairment and hyposmia. LRRK2 p.G2019S-associated PD is a more benign progression than idiopathic PD as patients with idiopathic PD need dopamine-replacement treatment earlier than patients with LRRK2 p.G2019S were more prone to drug-induced dyskinesia. The risk of PD for a person who inherits the LRRK2 p.G2019S mutation was 28% at age 59 years, 51% at 69 years, and 74% at 79 years [43].

Patients should be counseled based on their ethnic group. For example, LRRK2 p.G2019S mutation is found in 10% of Ashkenazi Jews with sporadic PD and in 4% of Portuguese patients with sporadic PD but in only 1% of white North Americans. In addition, patients should be aware that the presence of a pathogenic mutation does not influence treatment choices, and the main benefit of testing is to improve diagnostic accuracy unless neuroprotective drugs are discovered.

## LRRK2 in the possible pathogenic mechanism of Parkinson’s disease (Figure 2)

### LRRK2 and α-synuclein

α-synuclein is a 14 kDa neuronal protein that is highly expressed in the brain. In normal neurons, α-synuclein is typically associated with synaptic vesicles at axon terminals [44]. Although the physiological role of α-synuclein is largely unclear, research showed that mice lacking α-synuclein displayed defective dopaminergic transmission, whereas overexpression of α-synuclein disrupted catecholamine release, suggesting that the α-synuclein may play a role in modulating neurotransmitter vesicle function [45].

**Figure 2:**
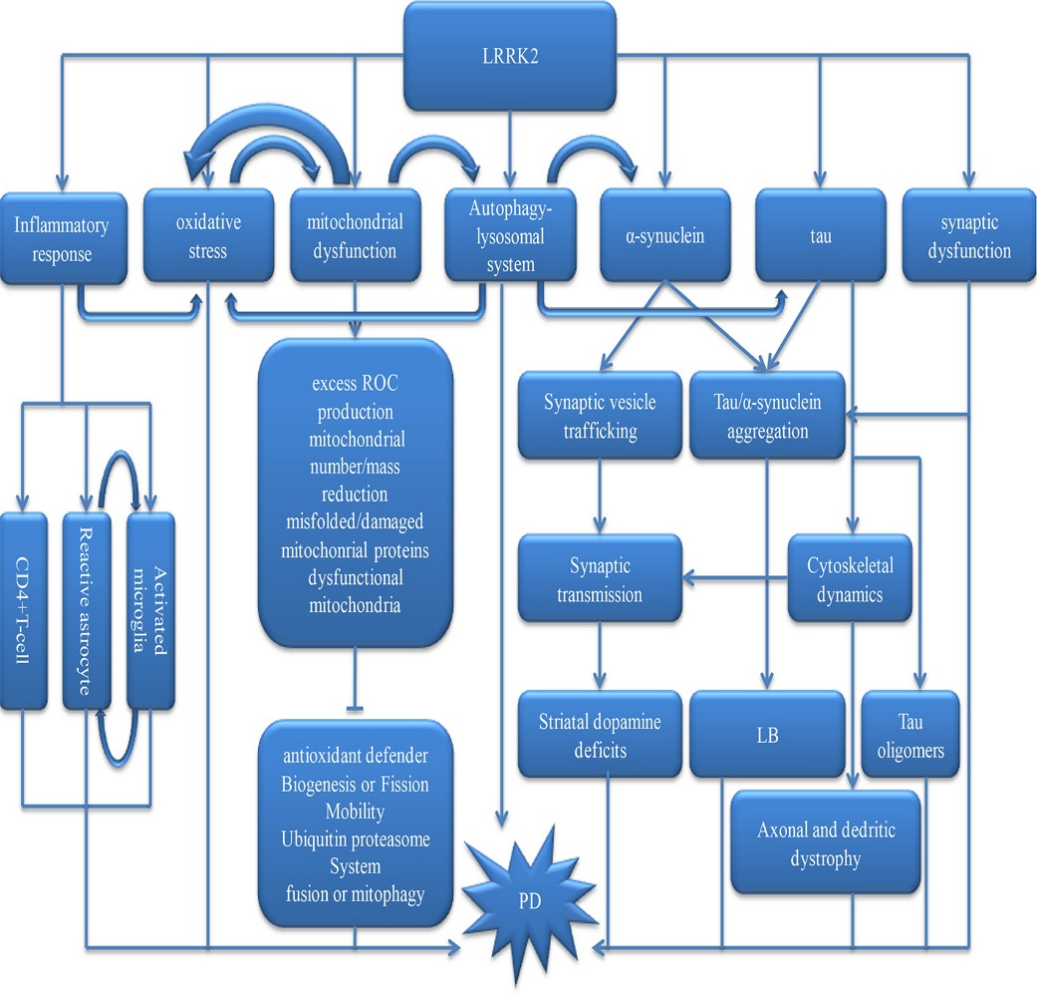
**LRRK2-mediated the phosphorylation of tubulin-associated tau LRRK2 interacts with tubulin-associated tau, resulting in the formation of a tripartite complex.** The interaction between LRRK2 and β-tubulin is mediated by the LRRK2 Roc domain and β-tubulin C termini. Tubulin-associated tau was phosphorylated by LRRK2. LRRK2 interacted directly with GSK-3β, and that His–GSK-3β bound to the kinase domain of LRRK2 (thick line). Phosphorylation of tau by GSK-3β was stimulated by LRRK2 (thin arrow). P.G2019S-LRRK2 had higher binding affinity for GSK-3β and showed much stronger stimulatory activity (thick line and arrow).

Postmortem analysis of PD patients with *LRRK2* mutations has reported neuropathology both with and without LBs. Further studies comparing *LRRK2* mutations cases with and without Lewy pathology are very valuable to elucidate the role of insoluble α-synuclein aggregates to neurodegeneration in these genetic forms of PD. Approximately 20–100% (mean = 60%) of SNCA-positive LBs contain LRRK2. The increase in striatal alpha-synuclein levels induce increased Lrrk2 mRNA levels which suggested that Lrrk2 and alpha-synuclein mRNA levels are possibly co-regulated. Either full-length LRRK2 or fragments containing its kinase domain has a significant capacity to phosphorylate recombinant alpha synuclein at serine 129. Moreover, the G2019S mutation in Lrrk2 has a significantly greater capacity than wild-type Lrrk2 to phosphorylate alpha synuclein [46]. Loss of LRRK2, on the other hand, seems to cause both accumulation and aggregation of SNCA. It has been reported that abundant α-synuclein pathology is present in the medulla, a region that is impacted early in disease [47, 48], suggesting that phosphorylation of α-synuclein is an early event in disease pathogenesis and might contribute to disease propagation [49]. Co-immunoprecipitation showed that endogenous LRRK2 and α-synuclein have interaction in cells, mouse and human brain tissue. This interaction has also been confirmed in over-expression studies in HEK-293 cells. It is also confirmed that the p.G2019S mutation did not alter the ability of LRRK2 to interact with α-synuclein in this model [50].

### LRRK2 and tau

Tau is a microtubule-associated protein which takes part in stabilizing microtubules by promoting their polymerization and suppressing their dissociation, and appears to have a stabilizing role during axonal outgrowth within the central nervous system [51, 52]. Similar to amyloid formation by a-synuclein, tau can aggregate and is deposited in neurofibrillary tangles (NFTs). Evidence suggests that NFTs is not the toxic species because neurons bearing NFT can survive for decades and there is no apparent causal relationship between apoptotic morphology and tau deposition. However, oligometric tau is toxic, because retinal degeneration is observed with tau expression alone in drosophila [53, 54].

**Figure 3:**
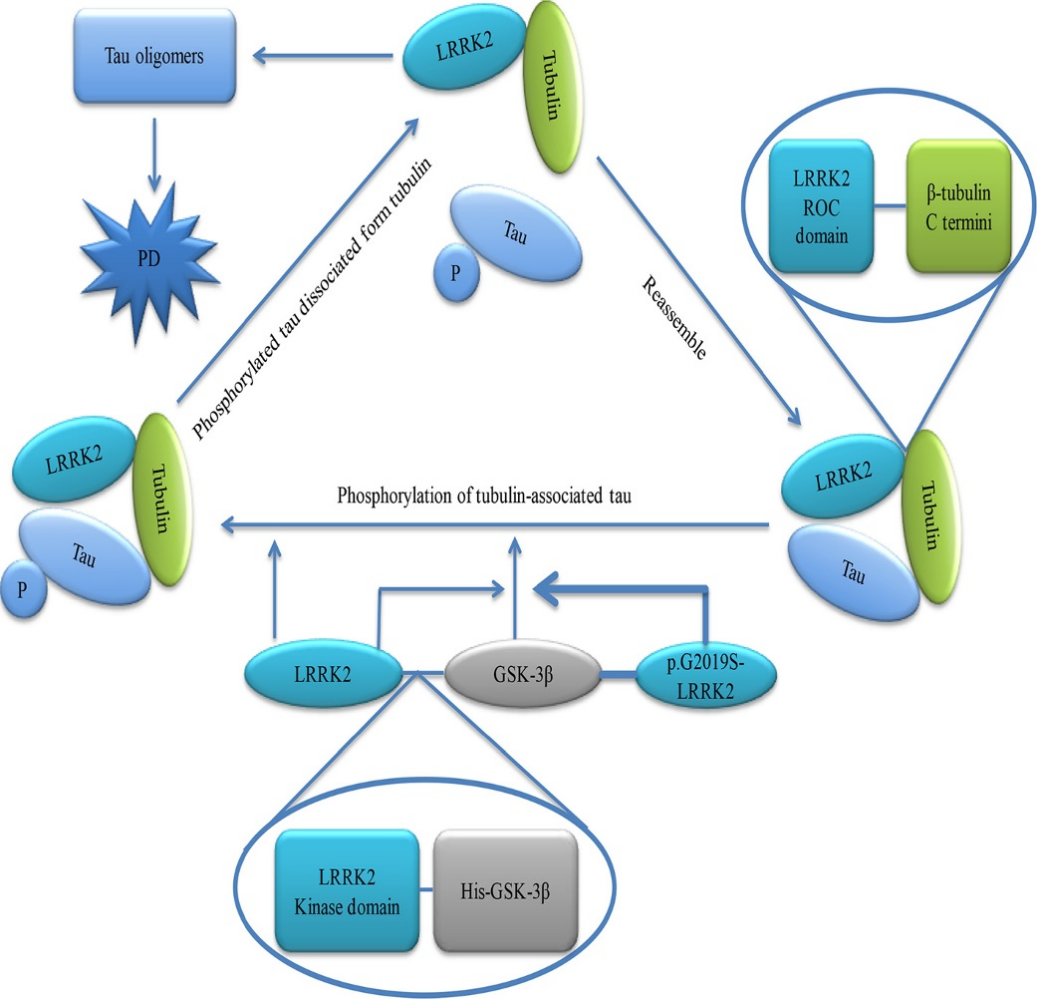
**The possible pathogenic mechanism links LRRK2 and Parkinson’s disease α-synuclein, tau, inflammatory response, oxidative stress, mitochondrial dysfunction, synaptic dysfunction and autophagy-lysosomal system all take part in the pathogenic of PD.** Open and filled arrows show positive (activating) and negative (suppressing) interactions, respectively.

A study showed that LRRK2 interacted and phosphorylated tubulin-associated tau but not free tau molecules [55]. Subsequently, they found that Thr181 was one of the phosphorylation target sites for LRRK2 in the presence of tubulin. This finding has been confirmed by a recent study which described a direct interaction between LRRK2 and β-tubulin [56]. In order to determine the effect of LRRK2 on the phosphorylation state of tau, researches have been conducted in an LRRK2-overexpressing SH-SY5Y cell clone, finding that phosphorylation of tau at Thr181 and Ser396 was significantly elevated in LRRK2-overexpressing cells and this increase was reduced by LRRK2 knockdown [57]. The phenomenon that endogenous LRRK2 and GSK-3β were coprecipitated by antibody against LRRK2 indicated that LRRK2 is able to interact with GSK-3β in human neuronal cells. Further research with an in vitro pull-down assay with recombinant proteins confirmed that LRRK2 interacted directly with GSK-3β, and that His–GSK-3β bound to the kinase domain of LRRK2. Furthermore, research conducted in vitro further confirmed that phosphorylation of tau by GSK-3β was significantly stimulated by LRRK2. Researchers also found that compared with wild-type-*LRRK2*, p.G2019S-*LRRK2* showed much stronger stimulatory activity for GSK-3β-mediated tau phosphorylation, and the stimulatory activities of other mutants such as p.R1441C-*LRRK2* and I2020T-*LRRK2* were equivalent to that of wild-type (WT) -LRRK2 [57] (Figure 3).

Mitogen-activated protein kinase (MAPK) activation induced by oxidative stress leads to tau phosphorylation which could negatively regulate its binding to microtubules. Studies showed that the binding of LRRK2 to tubulins would be enhanced by LRRK2 inhibitor LRRK2-IN1 in cells. Co-incubation of LRRK2 with microtubules increased the LRRK2 GTPase activity in a cell-free assay; however, destabilization of microtubules causes a decreased LRRK2 kinase activity [58].

Neurodegenerative diseases with prominent tau pathology in the central nervous system (CNS), within the neuronal compartment and also glial cells termed tauopathy. In tauopathies, the soluble tau protein detached from microtubules increases the concentration of soluble tau, enhance its propensity to aggregate. Although some tauopathies are characterized by parkinsonism, which is partially responsive to levodopa; some are characterized by FTD; others are characterized by a motor neuron disorder phenotype. For instance, PSP which belongs to Parkinson’s Plus Syndromes is a tauopathy with predominant tau pathology and prominent parkinsonism. corticobasal degeneration (CBD) is also part of Parkinson’s Plus Syndromes. Microscopically there are numerous of tau-immunoreactive astrocytic plaques which have not been seen in other tauopathies [59].

### LRRK2 and inflammatory response

Emerging evidences indicated that inflammation was closely linked with pathophysiology and aetiology of neurodegenerative diseases and the triggering factors may be pathogens, the dysregulation of inflammatory pathways, protein aggregates and primary inflammatory responses in neurons. The current hypothesis is that infammation may play a role in the prodromal stages of the disease even preceding the onset of PD. Blocking the early phase of neuroinflammation by minocycline or the application of non-steroidal anti-inflammatory drugs, cyclooxygenase-2 (COX-2), soluble tumor necrosis factor or IL-1beta inhibitors attenuates the PD-like disease process which gave strong supporting to this hypothesis [60–63]. Although the exact relationship between inflammation and neurodegeneration remains largely unknown, the description of microglias, astrocytes, and lymphocytes in animal models of dopamine (DA) neurodegeneration and post-mortem samples in PD patients would be helpful to solve this problem.

LRRK2 may contribute to PD pathogenesis by directly triggering dysfunction in immune cells. The expression of LRRK2 is not only limited to neural cell line but also can be expanded to antigen-presenting cells including microglia, monocytes and B cells and is involved in the immune responses to pathogens [64, 65]. A robust induction of LRRK2 protein has been observed in microglia cells of mouse substantia nigra pars compacta (SNpc) or striatum after lipopolysaccharide (LPS)-induced inflammation [66]. However, in LRRK2 p.R1441G knock-in mice, LPS-activated microglia cells showed elevated expression of pro-inflammatory cytokines and reduced expression of anti-inflammatory cytokines compared with wild-type control microglia cells [67]. Moreover, primary cortical neurons tested with conditioned medium from LPS-stimulated LRRK2 p.R1441G microglia showed increased neuronal death compared with medium from wild-type LRRK2 microglia. Taken together, these data indicate that LRRK2 is involved in inflammatory reaction process, and that LRRK2 p.R1441G mutation might push microglia toward a pro-inflammatory state, which results in more serious inflammation and consequent neurodegeneration in patients with PD. LRRK2 p.R1441G and iPD fibroblasts displayed similar features in most assays whereas LRRK2 p.G2019S showed milder alterations. In LPS-responsive human embryonic kidney (HEK293T) cells, the overexpression of p.G2019S increased basal and LPS-induced levels of phosphorylated p38 and JNK. This suggested LRRK2 to be a positive regulator of inflammation in murine microglia, and *LRRK2* mutations may alter the microenvironment of the brain to favor neuroinflammation [68]. The enhanced kinase activity of G2019S is crucial for the neurotoxicity of this mutant. However, whether the kinase activity of LRRK2 is linked to the regulation of inflammatory responses is questionable.

Although the exact mechanism remains yet to be carefully explored, considerable progress supported a role for LRRK2 as regulator of inflammation by modulating transcription factors and inducing inflammatory genes. Inhibit or down-regulate LRRK2 could attenuate levels of inflammatory mediators including TNF-α, IL-1β, IL-6 and inducible nitric oxide synthase within the murine microglia cells [66, 68]. Notably, *LRRK2* knockdown microglia exhibited a significant reduction of nuclear factor kappa B transcriptional activity which is essential for induction of pro-inflammatory cytokines. Moreover, mechanistic studies showed that LRRK2 was a potent negative regulator of nuclear factor of activated T cells (NFAT) transcription factors, a class of molecules involved in immune cells [69]. These data may suggest that *LRRK2* mutations may be connected with excessive and deficient controlled immune responses, which a cause neuronal dysfunction and increase neuronal vulnerability to degeneration.

### LRRK2 and oxidative stress

Oxidative stress can be termed as a condition in which the cellular antioxidant defense mechanisms fails to keep the level of reactive oxygen species (ROS) within the normal range [70]. The reason may be due to either an overproduction of reactive free radicals or a failure of cell-buffering mechanisms [71]. Evidences from postmortem studies and vivo observations have indicated that oxidative stress is one of the most common causes to the pathogenesis of PD.

Compared with or without oxidative stress, cells over-expressing *LRRK2* WT and p.G2019S exhibited quite different percentages of cell death. The percentages are much higher in cells under oxidative stress. These data indicated that combination of either *LRRK2* WT or p.G2019S expression with oxidative stress synergistically increased cell death with the strongest effect being observed with over-expression of p.G2019S [72]. One possibility to explain the synergistic neurotoxic effect is the potential bystander effect. Bystander effect could be explained as factors secreted from p.G2019S-expressing cells under oxidative stress impair other neighboring untransfected cells. With regard to the exact mechanism of synergistic effect, researchers measured intracellular ROS using the 2′,7′-dichlorofluorescin diacetate assay, suggesting that LRRK2 can increase intracellular ROS level and that the p.G2019S mutation might enhance this effect, which was intensified by external oxidative stress. P.G2019S has been reported to increase kinase activity of LRRK2 WT protein as a cause of neurotoxicity enhancement. However, data suggests that, other domain(s) of LRRK2 can also partially contribute to the neurotoxicity but kinase domain is a predominant manager. Fortunately, this neurotoxicity could be at least partially rescued by DJ-1 or the ERK inhibitor treatment, which could be a therapeutic approach for us to choose [72].

Recently, further research conducted in a yeast model for full-length LRRK2 studies indicated that LRRK2 confers cellular protection during oxidative stress depending on mitochondrial function and endocytosis [73]. Since in recent years a connection of LRRK2 with mitochondria has been well demonstrated, further research has been done to determine whether functional mitochondria is determinate for LRRK2 response to oxidative stress. The answer is affirmative. But this effect was not observed for PD-mutants G2019S and p.R1441C or in the absence of the kinase activity and the WD40 repeat domain. For the PD-mutants, endocytic defects were also observed having a negative impact on LRRK2-mediated protection against hydrogen peroxide. In addition, another experiment showed that Peroxiredoxin 3 protects against mutant LRRK2-induced oxidative stress related macromolecular damage [74]. Moreover, *LRRK2* mutant-mediated inhibition of peroxidase activity could elevate oxidant level and potentially oxidative stress [75].

### LRRK2 and mitochondrial dysfunction

Mitochondrial dysfunction is indirectly linked to parkinsonism. There has been clear evidence presented for a high burden of deleted mitochondrial DNA (mtDNA) within the substantia nigra neurons in individuals with PD [76]. There are more compelling evidences which come from mice possessing conditional knockout of mitochondrial transcription factor A in DA neurons, showing reduced mtDNA expression which in turn leads to a parkinsonism phenotype characterized by adult onset of a progressive impairment of motor function accompanied by formation of intraneuronal inclusions and DA nerve cell death [77]. Further evidence for mitochondrial involvement in the pathogenesis of PD is supported by post-mortem biochemical studies in human beings with idiopathic PD.

Experiment conducted in primary cultured cortical neurons found that the localization of endogenous LRRK2 was widely distributed in the cytosol and partially co-localized with Cyto C (marker for mitochondria). Both LRRK2 WT and LRRK2 p.G2019S could induce mitochondrial fragmentation after transfection, whereas the latter going with more severe effect, which was related to cell death which is consistent with earlier statements [78, 79]. This result shows that LRRK2 is likely to be an important regulator of mitochondrial fission/fusion dynamics. Moreover, endogenous LRRK2 was further confirmed to partially co-localize with mitochondrial fission Dynamin like protein 1 (DLP1) in cortical neurons which suggests that DLP1 is very likely to be involved in LRRK2-induced mitochondrial fission [80]. It has also been reported that reduced mitochondrial membrane potential and total intracellular ATP levels accompanied by increased mitochondrial elongation and interconnectivity may be exhibited in PD patients with the p.G2019S mutation [81]. Similarly, reduced cell survival accompanying with mitochondrial dysfunction could also be observed in C. elegans with knock-down of lrk-1 (the endogenous ortholog of LRRK2) [82]. And human *LRRK2* expressed in C.elegans improved its survival under the treatment of the mitochondrial complex inhibitor such as rotenone or paraquat [83]. All of the data proved that LRRK2 may play an important part in regulating mitochondrial homeostasis [84]. In addition, mutations in LRRK2 could break mitochondrial function and increase the vulnerability of induced pluripotent stem cell (iPSC)-derived neural cells for patients with oxidative stress. There were higher levels of mtDNA damage in iPSC-derived neural cells from patients carrying LRRK2 p.G2019S mutation, or at-risk individuals carrying the LRRK2 p.R1441C mutation, than in cells from healthy subjects who do not carry *LRRK2* mutations since mtDNA damage can compromise mitochondrial function. However, mtDNA damage would not be detected in differentiated neuroprogenitor and neural cells after the repair by zinc finger nuclease of the LRRK2 p.G2019S mutation. Those evidences all suggest the link between *LRRK2* mutations and mtDNA damage that could be used for examining pathogenic mechanisms and screening therapeutic strategies [85, 86].

### LRRK2 and synaptic dysfunction

Accumulating evidences suggest that synaptic dysfunction might be an early event in neurodegeneration and has a close connection with PD. It has also been demonstrated that overexpression of mutant *LRRK2* and LRRK2 cDNAs containing PD-associated mutations in primary cortical cultures could result in gradual neurite injury and retraction which is accompanied by induction of neuritic autophagy and can lead to neurite shortening [87, 88]. This suggests that early events in the neurodegenerative cascade downstream of mutant LRRK2 is likely to target the neuritic/synaptic compartment.

Endogenous and overexpressed LRRK2 immunoreactivity has been located to various membrane containing structures in neurons, including Golgi and endoplasmic reticulum, lysosomes, mitochondria, the plasma membrane, as well as a partial localization to synaptic vesicles, structures of the endocytic pathway, including lipid rafts, caveolar necks, clathrin-coated endosomes and multivesicular endosomes, and microtubules [89–91]. A research conducted by measuring evoked excitatory postsynaptic current (EPSC) in pairs of synaptically connected pyramidal cortical neurons to test whether *LRRK2* silencing could affect presynaptic mechanism, found that EPSC amplitude in postsynaptic neurons increased more than twofold compared with that measured in control pairs. Furthermore, followed by a presynaptic trigger, siLRRK2 pairs exhibited a higher probability to generate an EPSC over the baseline. In addition, another source of data suggested that LRRK2 could modulate vesicle motility inside the presynaptic bouton and control synaptic vesicle distribution within the presynaptic bouton without affecting readily releasable pool (RRP) size [92]. An experience with the purpose of determining whether LRRK2 is involved in synaptic vesicle endocytosis in Drosophila, showed that *LRRK2* mutants fail to maintain release during intense stimulation and a defect often is observed in mutants with reduced synaptic vesicle endocytosis. However, the defect in synaptic vesicle formation in *LRRK2* mutants is neither caused by reduced vesicle fusion during stimulation nor major morphological changes at the neuromuscular junctions (NMJ). EndophilinA (EndoA) is an evolutionary conserved protein which plays a major role in membranes tabulation, vesicle formation and facilitating vesicle uncoating in order to regulate synaptic vesicle endocytosis in vivo. By this token, the EndoA dysfunction results in very severe defects in synaptic vesicle endocytosis [93]. Moreover, evidence has indicated that EndoA is a target of LRRK2 kinase activity in vitro, and one conserved site, serine 75(S75), is a target of LRRK2-dependent phosphorylation which may inhibit membrane tubulation in vitro [92]. There were also animal experiments showing that the expression of the EndoA S75 phosphomutants harbor synaptic vesicle recycling deficits that parallel the endocytic defect. Further research showed that both increased and decreased LRRK-dependent EndoA phosphorylation impedes synaptic vesicle endocytosis. In addition, another recent study proposed that autophagy may play an compensatory or deleterious role in shaping synaptic structure/function in PD [94].

### LRRK2 and autophagy-lysosomal system

Lysosomes are dynamic acidic organelles which could degrade intracellular components through several degradation pathways, including endocytosis, phagocytosis, and autophagy by hydrolytic enzymes [95, 96]. Postmortem brain samples from patients with idiopathic PD and toxic and genetic rodent models of PD exhibited a reduced number of intraneuronal lysosomes, decreased levels of lysosomal-associated proteins and accumulation of undegraded auto-phagosomes [97, 98]. In addition, there were reports that impaired lysosomal-mediated clearance of autophagosomes is shown in cultured dopaminergic neurons generated from reprogrammed iPSCs which is derived from skin fibroblasts of sporadic and genetic PD patients [99]. Mechanistic studies revealed that PD-linked lysosomal deficiency contributed to the impairment of autophagy before cell death in almost overall dopaminergic neurodegeneration [100]. Moreover, by knocking-out autophagy related 7 gene which is essential to autophagy within catecholaminergic neurons in mice, the results showed decreased striatal dopamine; abnormal presynaptic neurotransmission; and age-dependent axonal morphologic alterations, motor deficits, and neurodegeneration [101–103]. As mentioned above, emerging evidences indicates that impairment of lysosomal function may contribute to the pathogenesis of PD.

Experiments found that LRRK2 is specifically located in membrane microdomains such as the neck of caveolae, microvilli/filopodia and in cytoplasmic puncta which correspond to multivesicular bodies (MVBs) and autophagic vacuoles (AVs). Although the link between the *LRRK2* mutations and lysosomal pathways has not been completely clear, recent studies conducted in cells and knock-out mice have implicated LRRK2 to be in charge of regulating the assembly of the cytoskeleton [104] and the lysosomal-autophagic pathway. The LRRK2 p.R1441C mutation induced autophagic stress characterized by the accumulation of MVBs, abnormal AVs and skein-like cellular lesions. P.R1441C mutation in LRRK2 showed increased density of autophagic vacuoles in the brain cortex. Finally, siRNA-mediated *LRRK2* knockdown showed a tendency to revert the negative influence of starvation-induced cell death when autophagy was inhibited [90, 105]. As to LRRK2 p.G2019S, there would be axonal spheroid inclusions, which are made of swollen lysosomes in neuronal cultures [87]. It could also cause neurite shortening in differentiated neuroblastoma cells involving active autophagy. Moreover, p.G2019S mutant fibroblasts exhibited higher autophagic activity levels which could trigger cell death with increasing apoptosis hallmarks. The exact evidence showed that the p.G2019S mutation induces autophagy via the phosphorylation of MAPK/ERK kinases and that inhibition of this exacerbated autophagy by a highly selective inhibitor of MEK1/2 reduces the sensitivity observed in p.G2019S mutant cells [106, 107]. In the human brain, WT LRRK2 is located in the endosomal-lysosomal compartment within morphologically altered neurons in neurodegenerative diseases, and is increased in cases with LB pathology [108]. As in Drosophila, LRRK2 is located in the membranes of late endosomes and lysosomes, which negatively regulates rab7-dependent perinuclear localization of lysosomes and normally interacts with the crucial mediator of late endosomal transport Rab7. However, mutant form of *LRRK2* promotes rab7-dependent perinuclear lysosome clustering which links endolysosomal dysfunction to the pathogenesis of LRRK2-mediated PD [109]. Thus, it appears that LRKK2 function as lysosomal protein trafficking and possibly by means of regulating the rearrangement of the cytoskeleton. In addition, evidences from an age-dependent analysis of LRRK2-/- kidneys indicated that *LRRK2* mutations may cause PD and cell death by impairing protein degradation pathways which suggests that LRRK2 is required for normal regulation of the autophagy-lysosomal pathway [110]. However, evidence for such regulation by LRRK2 in the brain is lacking. Macroautophagy could be stimulated in the absence of any alteration in the translational targets of mTORC1 by using a LRRK2 pharmacologic inhibitor, suggesting that LRRK2 regulates autophagic vesicle formation independent of canonical mTORC1 signaling [111]. Additional data indicated that a lysosomal NAADP receptor such as TPC2 may be a target for LRRK2, followed by calcium release from lysosomes, leading to activation of a CaMKK/AMPK pathway to induce autophagy [112]. Moreover, A decrease in autophagic flux, which in conjunction with increases in p62 protein, autophagosome, and lipid droplets and is consistent with impaired autophagic clearance has recently also been described in human dopaminergic neurons derived from induced pluripotent stem cells from G2019S mutant LRRK2, but not control patients, after long-term culture [99]. At the moment, it remains unclear whether the *LRRK2* mutations are able to induce an increase or a decrease in the autophagic flux; consequently, it is not clear whether there is a positive or a negative contribution of LRRK2 in the control of autophagy. All in all, great promise stands that autophagy regulation may eventually become a useful and effective way to prevent and treat PD, and the research of LRRK2 may be a valuable approach.

## LRRK2 as a therapeutic target for parkinsonism

For the treatment of Parkinson’s disease, comprehensive treatment should be taken, such as drug treatment, surgical therapy, rehabilitation therapy, psychological therapy and so on. Among them, drug treatment is the first choice and the main treatment. For example, deep brain stimulation (DBS) of the subthalamic nucleus (STN) is an established therapy for advanced PD and the STN-DBS outcomes were not influenced by the LRRK2 p.G2019S mutation [113]. However, all the treatments just cannot stop the process of PD, needless to say to cure. The primary goals of all the therapies are to relieve the motor and non-motor symptoms of PD. Therefore, the requirement for new drugs to reverse, prevent, or slow down the progress of PD is one of the major goals for PD research.

Since LRRK2 is a kinase, there has been a hypothesis that targeting kinase activity might be a therapeutic approach for familial PD. Furthermore, as LRRK2-related PD and sporadic PD have same clinical characters, it might be deduced that targeting kinase activity might also be effective in treating idiopathic form of the disease (Table 2).

**Table 2:** **LRRK2 as a therapeutic target for parkinsonism**

		Model systems	Possible outcome	Clinical results
**Kinase inhibitor**	Staurosporine	both in vitro and in vivo	Inhibits LRRK2 autophosphorylation or LRRK2-mediated phosphorylation of myelin basic protein. Disrupts LRRK2 dimers.	-
	Sunitinib	in vitro	Inhibits LRRK2-mediated phosphorylation of LRRKtide and Nictide. The LRRK2 A2016T mutant is resistant to this compound.	-
	H-1152	in vitro	Inhibits LRRK2-mediated phosphorylation of LRRKtide and Nictide. The LRRK2 A2017T mutant is resistant to this compound.	-
	Indirubin-3’-monooxime	both in vitro and in vivo	Inhibits LRRK2 autophosphorylation and MBP and 4E-BP phosphorylation.	-
	Sorafenib	C. elegans and Drosophila models	Inhibits LRRK2 autophosphorylation and LRRK2-mediated MBP phosphorylation.	-
	GW5074	C. elegans and Drosophila models	Inhibits LRRK2 autophosphorylation and MBP and 4E-BP phosphorylation.	-
	CZC-25146	rodent model	Inhibits mutant LRRK2-mediated toxicity in primary rodent and human neurons.	-
	CZC-54252	rodent model	Inhibits mutant LRRK2-mediated toxicity in human neurons.	-
	LRRK2-IN-1	rodent model	Induces loss of 14-3-3 binding to LRRK2. Promotes dephosphorylation of Ser910 and Ser935 on LRRK2.	-
**GTPase modulator**	ArfGAP1	Drosophila model	Enhances both WT and mutant LRRK2 GTP hydrolysis.	-
**Synthetic agent**	diapocynin	rodent model	-	protect neurobehavioral function

Protein kinases act as a therapeutic target for parkinsonism because of their prominent roles in various types of diseases including neurodegenerative diseases. Several small-molecule kinase inhibitors such as imatinib (Gleevec), sorafenib (Nexavar), sunitinib (Sutent), and rapamycin (Sirolimus) have been admitted by the US Food and Drug Administration and are available on the market. Since directly monitoring LRRK2 activity in cells would be advantageous for the development of LRRK2 inhibitors, recent studies demonstrated that a monoclonal anti-LRRK2 antibody directed against the activation segment binds less efficiently to native LRRK2 protein in the presence of ATP-competitive LRRK2 inhibitors and induce a hypothesize that the antibody preferentially binds to the active conformation of LRRK2 under native conditions [114]. Kinases often take part in signaling cascades, and upstream and downstream effectors of LRRK2 are also attractive targets for pharmacological therapy for PD. Alternative kinase-related signaling pathways that could be intervened by drugs include protein kinase Cd, the MLK–c-Jun N-terminal kinase signaling cascade, and AKT/protein kinase B signaling cascade, which are all involved in apoptosis [115, 116]. The selectivity of most kinase inhibitors is incomplete, as most of them would inhibit other kinases depending on the structural similarity in this region of the kinase. However, LRRK2-IN1 and CZC-25146 have better selectivity than other kinases for LRRK2 as report recently [117, 118]. Experiment conducted in LRRK2 p.G2019S cells with a doxorubicin inducible neuritic phenotype showing a reversed phenotype after adding a specific and potent kinase inhibitor, IN-1. Moreover, LRRK2 p.G2019S-mediated blunting of neurite extension is reversed by Lentivirus-mediated transfer of LRRK2 p.G2019S allele-specific small hairpin RNA [119]. Another similar study conducted in transgenic C. elegans which expressing human p.R1441C- and p.G2019S-LRRK2 showed that TTT-3002 and LRRK2-IN1 rescued the behavioral deficit characteristic of dopaminergic impairment. However, TTT-3002 and LRRK2-IN1 were ineffective against the p.A2016T mutation of *LRRK2*, suggesting that they targeting *LRRK2* specifically [120]. Several lines of evidence in vitro and in vivo both suggested a high therapeutic potential of CEP1347 as it showed neuroprotective effects in a variety of PD and neurodegenerative models. However, when it comes to the clinic, therapeutic efficacy was not turned out well [121]. This example remind us to notice the differences from bench to bedside and the potential pitfalls of evaluating kinase inhibitors as neuroprotective agents in PD [122].

Other than LRRK2 kinase inhibition, there’s also other possible target such as modulation of LRRK2 GTPase activity. Fameshift LRRK2 could interact with other different proteins by binding of GTP to it. This therapy might be especially effective for p.R1441C- and p.Y1699C-mutant *LRRK2* because the data on LRRK2 p.R1441C and p.Y1699C mutation having lower GTPase activity which suggests that the GTP-bound form is associated with disease. Therefore, blocking the GTP-binding pocket of ROC or stimulation of GTPase activity to limit a pathogenic interaction could be a advisable therapeutic target for LRRK2-associated PD [123].

Moreover, a study carried by Dranka et al. indicated that diapocynin (5,5’-dehydrodiacetovanillone) which is a synthetic compound conversed form Apocynin (4-hydroxy-3-methoxyacetophenone) could prevents early PD symptoms in the LRRK2 p.R1441G transgenic mouse. After treatment with diapocynin, the improvement of performance on the pole test and Rotor-Rod in the LRRK2 p.R1441G mice demonstrated that diapocynin is a viable agent for protection of neurobehavioral function [124]. Finally, it has been shown that deletion of N-terminal LRR portion or WD40 of p.G2019S or p.R1441C rescued LRRK2-induced toxicity in neuronal cells because these protein-protein interaction domains with no catalytic activity may play a role in mediating the cytotoxicity induced by LRRK2 kinase activity [125]. Inhibit peptide in blocking the binding of proteins which involved in mediating LRRK2-induced cytotoxicity may also be a point of therapeutic intervention as has been done for other globular proteins that interact.

## Concluding remarks

PD is a devastating neurodegenerative disorder, but an exact understanding of disease etiology and a preventive therapeutic approach are still elusive. Identification of mutations in *LRRK2* that cause autosomal-dominant parkinsonism closely resembling idiopathic disease represents a new chapter in PD research. LRRK2, also known as dardarin, is a large protein of unknown functions, because no physiological substrates have been identified so far. Therefore, future studies should be conducted to explore a better understanding of the function of dardarin and determine the identification of possible interactors that could have an effect on disease features which are definitively necessary to elucidate the biochemical pathway underlying PD. What’s more, a full sequence analysis would be the most preferred mutation screening method and focusing on the exons encoding the last five domains of dardarin could be a more effective screening approach. The variability in pathology indicates a link between different forms of neurodegeneration. However, exactly how dysfunction of LRRK2 leads to PD is unclear. We reviewed the possible pathogenic mechanisms link LRRK2 with PD from several aspects.

Although much has been learned about LRRK2 in the last few years, many problems remain unanswered. For example, what exact functional effect do LRRK2 PD-associated mutations have on neurons? *LRRK2* expression has been suggested to cause loss of neuronal viability and it also has a strong effect on the length of neurites on these cells, whether this is true toxicity or not is unclear. Why are dopaminergic neurons particularly sensitive to *LRRK2* mutations when the protein has a widespread expression pattern? Moreover, it remains to be seen whether LRRK2 is essential to dopaminergic neurotransmission.

## Abbreviations

Aβ42: Amyloid-beta 42
AD: Autosomal dominant
ANK: Ankyrin
AP: Atypical parkinsonism
ARM: Armadillo
AVs: Autophagic vacuoles
CBGD: Corticobasal ganglionic degeneration
CBD: Corticobasal degeneration
CNS: Central nervous system
COR: Carboxy- terminal of Roc
COX-2: Cyclooxygenase-2
CSF: Cerebrospinal Fluid
DA: Dopamine
DBS: Deep brain stimulation
DLP1: Dynamin like protein 1
EndoA: EndophilinA
EPSC: Excitatory postsynaptic current
FTD: Frontotemporal dementia
GCIs: Glial cytoplasmic inclusions
GFP: Green fluorescent protein
iPSC: Induced pluripotent stem cell
LB: Lewy bodies
LBD: Lewy body disease
LRR: Leucine-rich repeats
LRRK2: Leucine-rich repeat kinase 2
MAPK: Mitogen-activated protein kinase
MSA: Multiple system atrophy
mtDNA: Mitochondrial DNA
MVBs: Multivesicular bodies
NFAT: Nuclear factor of activated T cells
NFTs: Neurofibrillary tangles
NMJ: Neuromuscular junctions
PET: Positron emission tomography
PD: Parkinson’s disease
PPA: Primary progressive aphasia
PSP: Progressive supranuclear palsy
Roc: Ras of complex
ROS: Reactive oxygen species
SNpc: Substantia nigra pars compacta
STN: Subthalamic nucleus
WT: Wild-type.

## References

[1] Kumari U, Tan EK (2009). LRRK2 in Parkinson’s disease: genetic and clinical studies from patients. FEBS J.

[2] Bardien S, Lesage S, Brice A, Carr J (2011). Genetic characteristics of leucine-rich repeat kinase 2 (LRRK2) associated Parkinson’s disease. Parkinsonism Relat Disord.

[3] Marin I (2008). Ancient origin of the Parkinson disease gene LRRK2. J Mol Evol.

[4] Gilsbach BK, Kortholt A (2014). Structural biology of the LRRK2 GTPase and kinase domains: implications for regulation. Front Mol Neurosci.

[5] Cardona F, Tormos-Perez M, Perez-Tur J (2014). Structural and functional in silico analysis of LRRK2 missense substitutions. Mol Biol Rep.

[6] Mata IF, Wedemeyer WJ, Farrer MJ, Taylor JP, Gallo KA (2006). LRRK2 in Parkinson’s disease: protein domains and functional insights. Trends Neurosci.

[7] Skibinski G, Nakamura K, Cookson MR, Finkbeiner S (2014). Mutant LRRK2 toxicity in neurons depends on LRRK2 levels and synuclein but not kinase activity or inclusion bodies. J Neurosci.

[8] Anand VS, Braithwaite SP (2009). LRRK2 in Parkinson’s disease: biochemical functions. FEBS J.

[9] Rideout HJ, Stefanis L (2014). The Neurobiology of LRRK2 and its Role in the pathogenesis of Parkinson’s disease. Neurochem Res.

[10] Illarioshkin SN, Shadrina MI, Slominsky PA, Bespalova EV, Zagorovskaya TB, Bagyeva G, Markova ED, Limborska SA, Ivanova-Smolenskaya IA (2007). A common leucine-rich repeat kinase 2 gene mutation in familial and sporadic Parkinson’s disease in Russia. Eur J Neurol.

[11] Luzon-Toro B, Rubio de la Torre E, Delgado A, Perez-Tur J, Hilfiker S (2007). Mechanistic insight into the dominant mode of the Parkinson’s disease-associated G2019S LRRK2 mutation. Hum Mol Genet.

[12] Liu M, Bender SA, Cuny GD, Sherman W, Glicksman M, Ray SS (2013). Type II kinase inhibitors show an unexpected inhibition mode against Parkinson’s disease-linked LRRK2 mutant G2019S. Biochemistry.

[13] Di Fonzo A, Wu-Chou YH, Lu CS, van Doeselaar M, Simons EJ, Rohe CF, Chang HC, Chen RS, Weng YH, Vanacore N, Breedveld GJ, Oostra BA, Bonifati V (2006). A common missense variant in the LRRK2 gene, Gly2385Arg, associated with Parkinson’s disease risk in Taiwan. Neurogenetics.

[14] Liu Z, Wang X, Yu Y, Li X, Wang T, Jiang H, Ren Q, Jiao Y, Sawa A, Moran T, Ross CA, Montell C, Smith WW (2008). A Drosophila model for LRRK2-linked parkinsonism. Proc Natl Acad Sci U S A.

[15] Ng CH, Mok SZ, Koh C, Ouyang X, Fivaz ML, Tan EK, Dawson VL, Dawson TM, Yu F, Lim KL (2009). Parkin protects against LRRK2 G2019S mutant-induced dopaminergic neurodegeneration in Drosophila. J Neurosci.

[16] Venderova K, Kabbach G, Abdel-Messih E, Zhang Y, Parks RJ, Imai Y, Gehrke S, Ngsee J, Lavoie MJ, Slack RS, Rao Y, Zhang Z, Lu B, Haque ME, Park DS (2009). Leucine-rich repeat kinase 2 interacts with parkin, DJ-1 and PINK-1 in a Drosophila melanogaster model of Parkinson’s disease. Hum Mol Genet.

[17] Saha S, Guillily MD, Ferree A, Lanceta J, Chan D, Ghosh J, Hsu CH, Segal L, Raghavan K, Matsumoto K, Hisamoto N, Kuwahara T, Iwatsubo T, Moore L, Goldstein L, Cookson M, Wolozin B (2009). LRRK2 modulates vulnerability to mitochondrial dysfunction in Caenorhabditis elegans. J Neurosci.

[18] Wolozin B, Saha S, Guillily M, Ferree A, Riley M (2008). Investigating convergent actions of genes linked to familial Parkinson’s disease. Neurodegener Dis.

[19] Li Y, Liu W, Oo TF, Wang L, Tang Y, Jackson-Lewis V, Zhou C, Geghman K, Bogdanov M, Przedborski S, Beal MF, Burke RE, Li C (2009). Mutant LRRK2(R1441G) BAC transgenic mice recapitulate cardinal features of Parkinson’s disease. Nat Neurosci.

[20] Lin X, Parisiadou L, Gu XL, Wang L, Shim H, Sun L, Xie C, Long CX, Yang WJ, Ding J, Chen ZZ, Gallant PE, Tao-Cheng JH, Rudow G, Troncoso JC, Liu Z, Li Z, Cai H (2009). Leucine-rich repeat kinase 2 regulates the progression of neuropathology induced by Parkinson’s-disease-related mutant alpha-synuclein. Neuron.

[21] Tong Y, Pisani A, Martella G, Karouani M, Yamaguchi H, Pothos EN, Shen J (2009). R1441C mutation in LRRK2 impairs dopaminergic neurotransmission in mice. Proc Natl Acad Sci U S A.

[22] Tong Y, Shen J (2012). Genetic analysis of Parkinson’s disease-linked leucine-rich repeat kinase 2. Biochem Soc Trans.

[23] Ross OA, Spanaki C, Griffith A, Lin CH, Kachergus J, Haugarvoll K, Latsoudis H, Plaitakis A, Ferreira JJ, Sampaio C, Bonifati V, Wu RM, Zabetian CP, Farrer MJ (2009). Haplotype analysis of Lrrk2 R1441H carriers with parkinsonism. Parkinsonism Relat Disord.

[24] Zhang Z, Burgunder JM, An X, Wu Y, Chen W, Zhang J, Wang Y, Xu Y, Gou Y, Yuan G, Mao X, Peng R (2009). LRRK2 R1628P variant is a risk factor of Parkinson’s disease among Han-Chinese from mainland China. Mov Disord.

[25] Pchelina SN, Ivanova ON, Emel’ianov AK, Iakimovskii AF (2011). Clinical features of LRRK2-associated Parkinson’s disease. Zh Nevrol Psikhiatr Im S S Korsakova.

[26] Santpere G, Ferrer I (2009). LRRK2 and neurodegeneration. Acta Neuropathol.

[27] Kalia LV, Kalia SK, McLean PJ, Lozano AM, Lang AE (2013). alpha-Synuclein oligomers and clinical implications for Parkinson disease. Ann Neurol.

[28] Marti-Masso JF, Ruiz-Martinez J, Bolano MJ, Ruiz I, Gorostidi A, Moreno F, Ferrer I, Lopez de Munain A (2009). Neuropathology of Parkinson’s disease with the R1441G mutation in LRRK2. Mov Disord.

[29] Hasegawa K, Stoessl AJ, Yokoyama T, Kowa H, Wszolek ZK, Yagishita S (2009). Familial parkinsonism: study of original Sagamihara PARK8 (I2020T) kindred with variable clinicopathologic outcomes. Parkinsonism Relat Disord.

[30] Trinh J, Amouri R, Duda JE, Morley JF, Read M, Donald A, Vilarino-Guell C, Thompson C, Szu Tu C, Gustavsson EK, Ben Sassi S, Hentati E, Zouari M, Farhat E, Nabli F, Hentati F, Farrer MJ (2014). A comparative study of Parkinson’s disease and leucine-rich repeat kinase 2 p.G2019S parkinsonism. Neurobiol Aging.

[31] Shadrina MI, Illarioshkin SN, Bagyeva G, Bespalova EV, Zagorodskaia TB, Slominskii PA, Markova ED, Kliushnikov SA, Limborskaia SA, Ivanova-Smolenskaia IA (2007). A PARK8 form of Parkinson’s disease: a mutational analysis of the LRRK2 gene in Russian population. Zh Nevrol Psikhiatr Im S S Korsakova.

[32] Gatto EM, Parisi V, Converso DP, Poderoso JJ, Carreras MC, Marti-Masso JF, Paisan-Ruiz C (2013). The LRRK2 G2019S mutation in a series of Argentinean patients with Parkinson’s disease: clinical and demographic characteristics. Neurosci Lett.

[33] Ozelius LJ, Foroud T, May S, Senthil G, Sandroni P, Low PA, Reich S, Colcher A, Stern MB, Ondo WG, Jankovic J, Huang N, Tanner CM, Novak P, Gilman S, Marshall FJ, Wooten GF, Chelimsky TC, Shults CW, North American Multiple System Atrophy Study Group (2007). G2019S mutation in the leucine-rich repeat kinase 2 gene is not associated with multiple system atrophy. Mov Disord.

[34] Haugarvoll K, Rademakers R, Kachergus JM, Nuytemans K, Ross OA, Gibson JM, Tan EK, Gaig C, Tolosa E, Goldwurm S, Guidi M, Riboldazzi G, Brown L, Walter U, Benecke R, Berg D, Gasser T, Theuns J, Pals P, Cras P, De Deyn PP, Engelborghs S, Pickut B, Uitti RJ, Foroud T, Nichols WC, Hagenah J, Klein C, Samii A, Zabetian CP (2008). Lrrk2 R1441C parkinsonism is clinically similar to sporadic Parkinson disease. Neurology.

[35] Bichler Z, Lim HC, Zeng L, Tan EK (2013). Non-motor and motor features in LRRK2 transgenic mice. PLoS One.

[36] Khan NL, Jain S, Lynch JM, Pavese N, Abou-Sleiman P, Holton JL, Healy DG, Gilks WP, Sweeney MG, Ganguly M, Gibbons V, Gandhi S, Vaughan J, Eunson LH, Katzenschlager R, Gayton J, Lennox G, Revesz T, Nicholl D, Bhatia KP, Quinn N, Brooks D, Lees AJ, Davis MB, Piccini P, Singleton AB, Wood NW (2005). Mutations in the gene LRRK2 encoding dardarin (PARK8) cause familial Parkinson’s disease: clinical, pathological, olfactory and functional imaging and genetic data. Brain.

[37] Santos-Reboucas CB, Abdalla CB, Baldi FJ, Martins PA, Correa JC, Goncalves AP, Cunha MS, Borges MB, Pereira JS, Laks J, Pimentel MM (2008). Co-occurrence of sporadic parkinsonism and late-onset Alzheimer’s disease in a Brazilian male with the LRRK2 p.G2019S mutation. Genet Test.

[38] Chen-Plotkin AS, Yuan W, Anderson C, McCarty Wood E, Hurtig HI, Clark CM, Miller BL, Lee VM, Trojanowski JQ, Grossman M, Van Deerlin VM (2008). Corticobasal syndrome and primary progressive aphasia as manifestations of LRRK2 gene mutations. Neurology.

[39] Shanker V, Groves M, Heiman G, Palmese C, Saunders-Pullman R, Ozelius L, Raymond D, Bressman S (2011). Mood and cognition in leucine-rich repeat kinase 2 G2019S Parkinson’s disease. Mov Disord.

[40] Brockmann K, Groger A, Di Santo A, Liepelt I, Schulte C, Klose U, Maetzler W, Hauser AK, Hilker R, Gomez-Mancilla B, Berg D, Gasser T (2011). Clinical and brain imaging characteristics in leucine-rich repeat kinase 2-associated PD and asymptomatic mutation carriers. Mov Disord.

[41] Tan EK, Skipper L, Chua E, Wong MC, Pavanni R, Bonnard C, Kolatkar P, Liu JJ (2006). Analysis of 14 LRRK2 mutations in Parkinson’s plus syndromes and late-onset Parkinson’s disease. Mov Disord.

[42] Sellbach AN, Boyle RS, Silburn PA, Mellick GD (2006). Parkinson’s disease and family history. Parkinsonism Relat Disord.

[43] Healy DG, Falchi M, O’Sullivan SS, Bonifati V, Durr A, Bressman S, Brice A, Aasly J, Zabetian CP, Goldwurm S, Ferreira JJ, Tolosa E, Kay DM, Klein C, Williams DR, Marras C, Lang AE, Wszolek ZK, Berciano J, Schapira AH, Lynch T, Bhatia KP, Gasser T, Lees AJ, Wood NW, International LRRK2 Consortium (2008). Phenotype, genotype, and worldwide genetic penetrance of LRRK2-associated Parkinson’s disease: a case–control study. Lancet Neurol.

[44] Tao-Cheng JH (2006). Activity-related redistribution of presynaptic proteins at the active zone. Neuroscience.

[45] Abeliovich A, Schmitz Y, Farinas I, Choi-Lundberg D, Ho WH, Castillo PE, Shinsky N, Verdugo JM, Armanini M, Ryan A, Hynes M, Phillips H, Sulzer D, Rosenthal A (2000). Mice lacking alpha-synuclein display functional deficits in the nigrostriatal dopamine system. Neuron.

[46] Qing H, Wong W, McGeer EG, McGeer PL (2009). Lrrk2 phosphorylates alpha synuclein at serine 129: Parkinson disease implications. Biochem Biophys Res Commun.

[47] Braak H, Del Tredici K, Rub U, de Vos RA, Jansen Steur EN, Braak E (2003). Staging of brain pathology related to sporadic Parkinson’s disease. Neurobiol Aging.

[48] Kingsbury AE, Bandopadhyay R, Silveira-Moriyama L, Ayling H, Kallis C, Sterlacci W, Maeir H, Poewe W, Lees AJ (2010). Brain stem pathology in Parkinson’s disease: an evaluation of the Braak staging model. Mov Disord.

[49] Kondo K, Obitsu S, Teshima R (2011). alpha-Synuclein aggregation and transmission are enhanced by leucine-rich repeat kinase 2 in human neuroblastoma SH-SY5Y cells. Biol Pharm Bull.

[50] Guerreiro PS, Huang Y, Gysbers A, Cheng D, Gai WP, Outeiro TF, Halliday GM (2013). LRRK2 interactions with alpha-synuclein in Parkinson’s disease brains and in cell models. J Mol Med (Berl).

[51] Andreadis A (2005). Tau gene alternative splicing: expression patterns, regulation and modulation of function in normal brain and neurodegenerative diseases. Biochim Biophys Acta.

[52] Ballatore C, Lee VM, Trojanowski JQ (2007). Tau-mediated neurodegeneration in Alzheimer’s disease and related disorders. Nat Rev Neurosci.

[53] Iqbal K, Alonso Adel C, Chen S, Chohan MO, El-Akkad E, Gong CX, Khatoon S, Li B, Liu F, Rahman A, Tanimukai H, Grundke-Iqbal I (2005). Tau pathology in Alzheimer disease and other tauopathies. Biochim Biophys Acta.

[54] Wittmann CW, Wszolek MF, Shulman JM, Salvaterra PM, Lewis J, Hutton M, Feany MB (2001). Tauopathy in Drosophila: neurodegeneration without neurofibrillary tangles. Science.

[55] Kawakami F, Yabata T, Ohta E, Maekawa T, Shimada N, Suzuki M, Maruyama H, Ichikawa T, Obata F (2012). LRRK2 phosphorylates tubulin-associated tau but not the free molecule: LRRK2-mediated regulation of the tau-tubulin association and neurite outgrowth. PLoS One.

[56] Law BM, Spain VA, Leinster VH, Chia R, Beilina A, Cho HJ, Taymans JM, Urban MK, Sancho RM, Ramirez MB, Biskup S, Baekelandt V, Cai H, Cookson MR, Berwick DC, Harvey K (2014). A direct interaction between leucine-rich repeat kinase 2 and specific beta-tubulin isoforms regulates tubulin acetylation. J Biol Chem.

[57] Kawakami F, Shimada N, Ohta E, Kagiya G, Kawashima R, Maekawa T, Maruyama H, Ichikawa T (2014). Leucine-rich repeat kinase 2 regulates tau phosphorylation through direct activation of glycogen synthase kinase-3beta. FEBS J.

[58] Caesar M, Zach S, Carlson CB, Brockmann K, Gasser T, Gillardon F (2013). Leucine-rich repeat kinase 2 functionally interacts with microtubules and kinase-dependently modulates cell migration. Neurobiol Dis.

[59] Ludolph AC, Kassubek J, Landwehrmeyer BG, Mandelkow E, Mandelkow EM, Burn DJ, Caparros-Lefebvre D, Frey KA, de Yebenes JG, Gasser T, Heutink P, H_glinger G, Jamrozik Z, Jellinger KA, Kazantsev A, Kretzschmar H, Lang AE, Litvan I, Lucas JJ, McGeer PL, Melquist S, Oertel W, Otto M, Paviour D, Reum T, Saint-Raymond A, Steele JC, Tolnay M, Tumani H, van Swieten JC (2009). Tauopathies with parkinsonism: clinical spectrum, neuropathologic basis, biological markers, and treatment options. Eur J Neurol.

[60] Wu DC, Jackson-Lewis V, Vila M, Tieu K, Teismann P, Vadseth C, Choi DK, Ischiropoulos H, Przedborski S (2002). Blockade of microglial activation is neuroprotective in the 1-methyl-4-phenyl-1,2,3,6-tetrahydropyridine mouse model of Parkinson disease. J Neurosci.

[61] Sanchez-Pernaute R, Ferree A, Cooper O, Yu M, Brownell AL, Isacson O (2004). Selective COX-2 inhibition prevents progressive dopamine neuron degeneration in a rat model of Parkinson’s disease. J Neuroinflammation.

[62] McCoy MK, Martinez TN, Ruhn KA, Szymkowski DE, Smith CG, Botterman BR, Tansey KE, Tansey MG (2006). Blocking soluble tumor necrosis factor signaling with dominant-negative tumor necrosis factor inhibitor attenuates loss of dopaminergic neurons in models of Parkinson’s disease. J Neurosci.

[63] Koprich JB, Reske-Nielsen C, Mithal P, Isacson O (2008). Neuroinflammation mediated by IL-1beta increases susceptibility of dopamine neurons to degeneration in an animal model of Parkinson’s disease. J Neuroinflammation.

[64] Hakimi M, Selvanantham T, Swinton E, Padmore RF, Tong Y, Kabbach G, Venderova K, Girardin SE, Bulman DE, Scherzer CR, LaVoie MJ, Gris D, Park DS, Angel JB, Shen J, Philpott DJ, Schlossmacher MG (2011). Parkinson’s disease-linked LRRK2 is expressed in circulating and tissue immune cells and upregulated following recognition of microbial structures. J Neural Transm.

[65] Gardet A, Benita Y, Li C, Sands BE, Ballester I, Stevens C, Korzenik JR, Rioux JD, Daly MJ, Xavier RJ, Podolsky DK (2010). LRRK2 is involved in the IFN-gamma response and host response to pathogens. J Immunol.

[66] Moehle MS, Webber PJ, Tse T, Sukar N, Standaert DG, DeSilva TM, Cowell RM, West AB (2012). LRRK2 inhibition attenuates microglial inflammatory responses. J Neurosci.

[67] Gillardon F, Schmid R, Draheim H (2012). Parkinson’s disease-linked leucine-rich repeat kinase 2(R1441G) mutation increases proinflammatory cytokine release from activated primary microglial cells and resultant neurotoxicity. Neuroscience.

[68] Kim B, Yang MS, Choi D, Kim JH, Kim HS, Seol W, Choi S, Jou I, Kim EY, Joe EH (2012). Impaired inflammatory responses in murine Lrrk2-knockdown brain microglia. PLoS One.

[69] Liu Z, Lee J, Krummey S, Lu W, Cai H, Lenardo MJ (2011). The kinase LRRK2 is a regulator of the transcription factor NFAT that modulates the severity of inflammatory bowel disease. Nat Immunol.

[70] Shulman JM, De Jager PL, Feany MB (2011). Parkinson’s disease: genetics and pathogenesis. Annu Rev Pathol.

[71] Yacoubian TA, Standaert DG (2009). Targets for neuroprotection in Parkinson’s disease. Biochim Biophys Acta.

[72] Heo HY, Park JM, Kim CH, Han BS, Kim KS, Seol W (2010). LRRK2 enhances oxidative stress-induced neurotoxicity via its kinase activity. Exp Cell Res.

[73] Pereira C, Miguel Martins L, Saraiva L (2014). LRRK2, but not pathogenic mutants, protects against HO stress depending on mitochondrial function and endocytosis in a yeast model. Biochim Biophys Acta.

[74] Angeles DC, Gan BH, Onstead L, Zhao Y, Lim KL, Dachsel J, Melrose H, Farrer M, Wszolek ZK, Dickson DW, Tan EK (2011). Mutations in LRRK2 increase phosphorylation of peroxiredoxin 3 exacerbating oxidative stress-induced neuronal death. Hum Mutat.

[75] Angeles DC, Ho P, Chua LL, Wang C, Yap YW, Ng C, Zhou ZD, Lim KL, Wszolek ZK, Wang HY, Tan EK (2014). Thiol peroxidases ameliorate LRRK2 mutant-induced mitochondrial and dopaminergic neuronal degeneration in Drosophila. Hum Mol Genet.

[76] Bender A, Krishnan KJ, Morris CM, Taylor GA, Reeve AK, Perry RH, Jaros E, Hersheson JS, Betts J, Klopstock T, Taylor RW, Turnbull DM (2006). High levels of mitochondrial DNA deletions in substantia nigra neurons in aging and Parkinson disease. Nat Genet.

[77] Ekstrand MI, Terzioglu M, Galter D, Zhu S, Hofstetter C, Lindqvist E, Thams S, Bergstrand A, Hansson FS, Trifunovic A, Hoffer B, Cullheim S, Mohammed AH, Olson L, Larsson NG (2007). Progressive parkinsonism in mice with respiratory-chain-deficient dopamine neurons. Proc Natl Acad Sci U S A.

[78] Iaccarino C, Crosio C, Vitale C, Sanna G, Carri MT, Barone P (2007). Apoptotic mechanisms in mutant LRRK2-mediated cell death. Hum Mol Genet.

[79] Cui J, Yu M, Niu J, Yue Z, Xu Z (2011). Expression of leucine-rich repeat kinase 2 (LRRK2) inhibits the processing of uMtCK to induce cell death in a cell culture model system. Biosci Rep.

[80] Niu J, Yu M, Wang C, Xu Z (2012). Leucine-rich repeat kinase 2 disturbs mitochondrial dynamics via Dynamin-like protein. J Neurochem.

[81] Mortiboys H, Johansen KK, Aasly JO, Bandmann O (2010). Mitochondrial impairment in patients with Parkinson disease with the G2019S mutation in LRRK2. Neurology.

[82] Yao C, El Khoury R, Wang W, Byrd TA, Pehek EA, Thacker C, Zhu X, Smith MA, Wilson-Delfosse AL, Chen SG (2010). LRRK2-mediated neurodegeneration and dysfunction of dopaminergic neurons in a Caenorhabditis elegans model of Parkinson’s disease. Neurobiol Dis.

[83] Todde V, Veenhuis M, van der Klei IJ (2009). Autophagy: principles and significance in health and disease. Biochim Biophys Acta.

[84] Liang CL, Wang TT, Luby-Phelps K, German DC (2007). Mitochondria mass is low in mouse substantia nigra dopamine neurons: implications for Parkinson’s disease. Exp Neurol.

[85] Sanders LH, Laganiere J, Cooper O, Mak SK, Vu BJ, Huang YA, Paschon DE, Vangipuram M, Sundararajan R, Urnov FD, Langston JW, Gregory PD, Zhang HS, Greenamyre JT, Isacson O, Schüle B (2014). LRRK2 mutations cause mitochondrial DNA damage in iPSC-derived neural cells from Parkinson’s disease patients: reversal by gene correction. Neurobiol Dis.

[86] Cooper O, Seo H, Andrabi S, Guardia-Laguarta C, Graziotto J, Sundberg M, McLean JR, Carrillo-Reid L, Xie Z, Osborn T, Hargus G, Deleidi M, Lawson T, Bogetofte H, Perez-Torres E, Clark L, Moskowitz C, Mazzulli J, Chen L, Volpicelli-Daley L, Romero N, Jiang H, Uitti RJ, Huang Z, Opala G, Scarffe LA, Dawson VL, Klein C, Feng J, Ross OA (2012). Pharmacological rescue of mitochondrial deficits in iPSC-derived neural cells from patients with familial Parkinson’s disease. Sci Transl Med.

[87] MacLeod D, Dowman J, Hammond R, Leete T, Inoue K, Abeliovich A (2006). The familial Parkinsonism gene LRRK2 regulates neurite process morphology. Neuron.

[88] Plowey ED, Cherra SJ, Liu YJ, Chu CT (2008). Role of autophagy in G2019S-LRRK2-associated neurite shortening in differentiated SH-SY5Y cells. J Neurochem.

[89] Hatano T, Kubo S, Imai S, Maeda M, Ishikawa K, Mizuno Y, Hattori N (2007). Leucine-rich repeat kinase 2 associates with lipid rafts. Hum Mol Genet.

[90] Alegre-Abarrategui J, Christian H, Lufino MM, Mutihac R, Venda LL, Ansorge O, Wade-Martins R (2009). LRRK2 regulates autophagic activity and localizes to specific membrane microdomains in a novel human genomic reporter cellular model. Hum Mol Genet.

[91] Biskup S, Moore DJ, Celsi F, Higashi S, West AB, Andrabi SA, Kurkinen K, Yu SW, Savitt JM, Waldvogel HJ, Faull RL, Emson PC, Torp R, Ottersen OP, Dawson TM, Dawson VL (2006). Localization of LRRK2 to membranous and vesicular structures in mammalian brain. Ann Neurol.

[92] Piccoli G, Condliffe SB, Bauer M, Giesert F, Boldt K, De Astis S, Meixner A, Sarioglu H, Vogt-Weisenhorn DM, Wurst W, Gloeckner CJ, Matteoli M, Sala C, Ueffing M (2011). LRRK2 controls synaptic vesicle storage and mobilization within the recycling pool. J Neurosci.

[93] Garcia J, Erickson K, Raji C, Lopez O, Newman A, Rosano C, Kuller L (2011). Physical activity is predictive of dementia but not mortality. Alzheimer’s and Dementia.

[94] Plowey ED, Chu CT (2011). Synaptic dysfunction in genetic models of Parkinson’s disease: a role for autophagy?. Neurobiol Dis.

[95] Luzio JP, Pryor PR, Bright NA (2007). Lysosomes: fusion and function. Nat Rev Mol Cell Biol.

[96] Lubke T, Lobel P, Sleat DE (2009). Proteomics of the lysosome. Biochim Biophys Acta.

[97] Alvarez-Erviti L, Rodriguez-Oroz MC, Cooper JM, Caballero C, Ferrer I, Obeso JA, Schapira AH (2010). Chaperone-mediated autophagy markers in Parkinson disease brains. Arch Neurol.

[98] Dehay B, Bove J, Rodriguez-Muela N, Perier C, Recasens A, Boya P, Vila M (2010). Pathogenic lysosomal depletion in Parkinson’s disease. J Neurosci.

[99] Sanchez-Danes A, Richaud-Patin Y, Carballo-Carbajal I, Jimenez-Delgado S, Caig C, Mora S, Di Guglielmo C, Ezquerra M, Patel B, Giralt A, Canals JM, Memo M, Alberch J, López-Barneo J, Vila M, Cuervo AM, Tolosa E, Consiglio A, Raya A (2012). Disease-specific phenotypes in dopamine neurons from human iPS-based models of genetic and sporadic Parkinson’s disease. EMBO Mol Med.

[100] Vila M, Bove J, Dehay B, Rodriguez-Muela N, Boya P (2011). Lysosomal membrane permeabilization in Parkinson disease. Autophagy.

[101] Friedman LG, Lachenmayer ML, Wang J, He L, Poulose SM, Komatsu M, Holstein GR, Yue Z (2012). Disrupted autophagy leads to dopaminergic axon and dendrite degeneration and promotes presynaptic accumulation of alpha-synuclein and LRRK2 in the brain. J Neurosci.

[102] Ahmed I, Liang Y, Schools S, Dawson VL, Dawson TM, Savitt JM (2012). Development and characterization of a new Parkinson’s disease model resulting from impaired autophagy. J Neurosci.

[103] Inoue K, Rispoli J, Kaphzan H, Klann E, Chen EI, Kim J, Komatsu M, Abeliovich A (2012). Macroautophagy deficiency mediates age-dependent neurodegeneration through a phospho-tau pathway. Mol Neurodegeneration.

[104] Parisiadou L, Xie C, Cho HJ, Lin X, Gu XL, Long CX, Lobbestael E, Baekelandt V, Taymans JM, Sun L, Cai H (2009). Phosphorylation of ezrin/radixin/moesin proteins by LRRK2 promotes the rearrangement of actin cytoskeleton in neuronal morphogenesis. J Neurosci.

[105] Boya P, Gonzalez-Polo RA, Casares N, Perfettini JL, Dessen P, Larochette N, Metivier D, Meley D, Souquere S, Yoshimori T, Pierron G, Codogno P, Kroemer G (2005). Inhibition of macroautophagy triggers apoptosis. Mol Cell Biol.

[106] Bravo-San Pedro JM, Niso-Santano M, Gomez-Sanchez R, Pizarro-Estrella E, Aiastui-Pujana A, Gorostidi A, Climent V, Lopez de Maturana R, Sanchez-Pernaute R, Lopez de Munain A, Fuentes JM, González-Polo RA (2013). The LRRK2 G2019S mutant exacerbates basal autophagy through activation of the MEK/ERK pathway. Cell Mol Life Sci.

[107] Bravo-San Pedro JM, Gomez-Sanchez R, Niso-Santano M, Pizarro-Estrella E, Aiastui-Pujana A, Gorostidi A, Climent V, Lopez de Maturana R, Sanchez-Pernaute R, Lopez de Munain A, Fuentes JM, González-Polo RA (2012). The MAPK1/3 pathway is essential for the deregulation of autophagy observed in G2019S LRRK2 mutant fibroblasts. Autophagy.

[108] Higashi S, Moore DJ, Yamamoto R, Minegishi M, Sato K, Togo T, Katsuse O, Uchikado H, Furukawa Y, Hino H, Kosaka K, Emson PC, Wada K, Dawson VL, Dawson TM, Arai H, Iseki E (2009). Abnormal localization of leucine-rich repeat kinase 2 to the endosomal-lysosomal compartment in lewy body disease. J Neuropathol Exp Neurol.

[109] Dodson MW, Zhang T, Jiang C, Chen S, Guo M (2012). Roles of the Drosophila LRRK2 homolog in Rab7-dependent lysosomal positioning. Hum Mol Genet.

[110] Tong Y, Giaime E, Yamaguchi H, Ichimura T, Liu Y, Si H, Cai H, Bonventre JV, Shen J (2012). Loss of leucine-rich repeat kinase 2 causes age-dependent bi-phasic alterations of the autophagy pathway. Mol Neurodegeneration.

[111] Manzoni C, Mamais A, Dihanich S, Abeti R, Soutar MP, Plun-Favreau H, Giunti P, Tooze SA, Bandopadhyay R, Lewis PA (1833). Inhibition of LRRK2 kinase activity stimulates macroautophagy. Biochim Biophys Acta.

[112] Gomez-Suaga P, Hilfiker S (2012). LRRK2 as a modulator of lysosomal calcium homeostasis with downstream effects on autophagy. Autophagy.

[113] Greenbaum L, Israeli-Korn SD, Cohen OS, Elincx-Benizri S, Yahalom G, Kozlova E, Strauss H, Molshatzki N, Inzelberg R, Spiegelmann R, Israel Z, Hassin-Baer S (2013). The LRRK2 G2019S mutation status does not affect the outcome of subthalamic stimulation in patients with Parkinson’s disease. Parkinsonism Relat Disord.

[114] Gillardon F, Kremmer E, Froehlich T, Ueffing M, Hengerer B, Gloeckner CJ (2013). ATP-competitive LRRK2 inhibitors interfere with monoclonal antibody binding to the kinase domain of LRRK2 under native conditions. a method to directly monitor the active conformation of LRRK2?. J Neurosci Methods.

[115] Burke RE (2007). Inhibition of mitogen-activated protein kinase and stimulation of Akt kinase signaling pathways: two approaches with therapeutic potential in the treatment of neurodegenerative disease. Pharmacol Ther.

[116] Cuny GD (2009). Kinase inhibitors as potential therapeutics for acute and chronic neurodegenerative conditions. Curr Pharm Des.

[117] Deng X, Dzamko N, Prescott A, Davies P, Liu Q, Yang Q, Lee JD, Patricelli MP, Nomanbhoy TK, Alessi DR, Gray NS (2011). Characterization of a selective inhibitor of the Parkinson’s disease kinase LRRK2. Nat Chem Biol.

[118] Ramsden N, Perrin J, Ren Z, Lee BD, Zinn N, Dawson VL, Tam D, Bova M, Lang M, Drewes G, Bantscheff M, Bard F, Dawson TM, Hopf C (2011). Chemoproteomics-based design of potent LRRK2-selective lead compounds that attenuate Parkinson’s disease-related toxicity in human neurons. ACS Chem Biol.

[119] Huang L, Shimoji M, Wang J, Shah S, Kamila S, Biehl ER, Lim S, Chang A, Maguire-Zeiss KA, Su X, Federoff HJ (2013). Development of inducible leucine-rich repeat kinase 2 (LRRK2) cell lines for therapeutics development in Parkinson’s disease. Neurotherapeutics.

[120] Yao C, Johnson WM, Gao Y, Wang W, Zhang J, Deak M, Alessi DR, Zhu X, Mieyal JJ, Roder H, Wilson-Delfosse AL, Chen SG (2013). Kinase inhibitors arrest neurodegeneration in cell and C. elegans models of LRRK2 toxicity. Hum Mol Genet.

[121] Parkinson Study Group PI (2007). Mixed lineage kinase inhibitor CEP-1347 fails to delay disability in early Parkinson disease. Neurology.

[122] Wang LH, Johnson EM (2008). Mixed lineage kinase inhibitor CEP-1347 fails to delay disability in early Parkinson disease. Neurology.

[123] Cookson MR (2010). The role of leucine-rich repeat kinase 2 (LRRK2) in Parkinson’s disease. Nat Rev Neurosci.

[124] Dranka BP, Gifford A, Ghosh A, Zielonka J, Joseph J, Kanthasamy AG, Kalyanaraman B (2013). Diapocynin prevents early Parkinson’s disease symptoms in the leucine-rich repeat kinase 2 (LRRK2R(1)(4)(4)(1)G) transgenic mouse. Neurosci Lett.

[125] Jorgensen ND, Peng Y, Ho CC, Rideout HJ, Petrey D, Liu P, Dauer WT (2009). The WD40 domain is required for LRRK2 neurotoxicity. PLoS One.

